# Atlanta Society of Medicine

**Published:** 1891-02

**Authors:** 


### ATLANTA SOCIETY OF MEDICINE.

The society met Tuesday evening, January 20th, and was called to order by the President, Dr. McRae. After roll-call by the Secretary, Dr. Childs,

### REPORTS OF CASES

were taken up.

Dr. Divine reported a very interesting case and presented pathological specimens.*

Dr. Duncan reported the following: I was called to see Mrs. J. B. on October 8th, 1890. I was informed that she was about six months advanced in first pregnancy. There was considerable œdema or general anasarca well marked in face, hands, feet and legs. Tongue clean, bowels regular, pulse 120. I suspected nephritis, but did not examine the urine at first, but made the following:

℞. Chloroform, 10 drops three or four times a day.
Kali sodidi, 5 grains after every meal.

After some two weeks there was some improvement, at which time there was a mere trace of albumen in urine. I saw her at intervals of four to ten days. She suffered some with pains in

*Dr. Divine hopes to publish this case in full when he gets his notes together, and receives a report of the microscopical examination, which is yet to be made.

arms, back and legs, which were not constant. She would leave off the treatment for a few days and every time there was an increase of pain and œdema. The appetite continued good, tongue clean and bowels regular, but the pulse continued rapid up to the time of her confinement, which took place January 20th, 1891. The labor was normal. I kept her partially under the influence of chloroform during the second stage of labor. When I removed the placenta there were firm contractions of the uterus. I had her husband to place his hand over the uterus and keep up gentle pressure, but notwithstanding this precaution there was considerable *post partum* hemorrhage. I introduced my hand into the uterus and removed the clots which were very large. There was immediate contraction. I gave some ergot, and there was no return of the trouble.

Dr. Jones reported the following: On December 25th I was called to a patient in labor, primipara, about twenty-two years of age, healthy and vigorous. Husband in excellent health and in good circumstances. After a normal labor of about five hours, the patient gave birth to a female child of about eight pounds weight, well developed and well formed, but possessing a broad single harelip, with cleft in palate extending completely back through both hard and soft palate.

The infant was put upon condensed milk and fed by hand with a spoon, taking its food with more ease than is usual in such cases, and has thriven well. The mother's breasts were bandaged, the secretion of milk suppressed, and she is now up. Will operate to restore lip as soon as the weather will permit.

Dr. Nicolson said he had been using the simple sutures in harelip operations and that he liked the change very much, as he thought the chances of producing a slough less under the use of sutures alone.

Dr. McRae did not believe that sufficient strength lay in the sutures alone, and thought that old cases especially needed the "harelip pins," mentioning a case recently operated in which they could not have been dispensed with.

Dr. Elkin showed the case of a negro man whose scrotum was enormously enlarged and thickened. Patient had stricture and perineal fistulæ. Besides the densely infiltrated scrotum there were several glazed fibrous-looking growths on perineum, which he and Dr. Hutchins considered keloidal. Dr. Elkin thought condition of scrotum was due to urinary infiltration, and remarked that the size was much decreased since some relief had been given to the stricture.

Dr. Hardon reported having lately tested the truth of the assertion that the sex of the child could be determined by the number of beats of the fœtal heart. The result did not sustain the theory.

Dr Hutchins said he wished to report a small case with a big name—onychomycosis. Patient a young man of 24. Several of the nails brittle, grayish and loose with epidermic accumulations beneath. Kept the nails cut from " bed." History of ringworm ten or twelve years ago ; nails probably infected from scratching. A peculiarity was that nails became affected successively and at intervals of two or three years. Scrapings from beneath diseased nails were mounted in liquor potassæ and the fungus of ringworm discovered. Diagnosis probably could not have been made otherwise. The case was mentioned only to show the diagnostic value of the microscope in certain obscure affections.

Dr. Childs reported the following case:

Mr. T, age 28, came to my office about three o'clock on the afternoon of January 12. He appeared nervous, excited, skin dry, lips indicated marked cyanosis; pulse 72, but weak; temperature, 96 degrees F.

He gave symptoms of La Grippe. Had taken twenty-five grains of acetanalid on morning of the day before, and on the morning after (the day I saw him) had taken fifteen grains more, and followed it an hour after with ten grains of same medicine. I prescribed quinine and phenacetine and a drink of whiskey. I saw him at three o'clock the following evening and found his temperature normal but pulse only 54 degrees. Decided he was suffering from overdose of ac.tanalid Continued the quinine and phenacetine and gave dose of Epsom salts. Have heard nothing more of the case.

Commenting on Dr. Childs' case, Dr. Nicolson mentioned similar cases of poisoning which he had seen.

Dr. McRae reported a case of repeated secondary hemorrhages from a wound of the wrist. One week before Christmas the patient fell and stuck a large splinter in his wrist. The splinter was extracted by Dr. Claude White, of Cartersville, and there being only slight hemorrhage he applied a simple antiseptic compress and dressing. Subsequently he had a more or less copious hemorrhage and the wound was again dressed by Dr. White. He then came to Atlanta and while walking around two weeks after the injury, there was a sudden gush of blood and quite a copious hemorrhage. I was called to see the case in consultation with Dr. Parks. We found the wound suppurating and extensive infiltration. Compression of radial artery seeming to control the hemorrhage, we ligated that artery just above the injury, Dr. L. P. Stephens and Mr. J. K. McKinnon, M. S., assisting; abundant antiseptic dressing applied. The patient had no further trouble until after a lapse of eight days, when he again had a copious hemorrhage. Dr. Parks was sick, and on reaching the patient I found him almost exsanguined. Bleeding being controlled by compression of brachial artery, ligated the brachial artery just above elbow and dressed both wounds antiseptically. Patient has had no further trouble.

